# Acute renal failure by rapidly progressive glomerulonephritis with IgA deposition in a patient concomitantly diagnosed with multibacillary Hansen's disease: a case report

**DOI:** 10.1590/2175-8239-JBN-2018-0056

**Published:** 2018-08-23

**Authors:** Yuri de Deus Mont'alverne Parente, Amanda Lopes de Castro, Flávio Bezerra de Araújo, André Costa Teixeira, Ítalo Criszostomo Lima, Elizabeth De Francesco Daher

**Affiliations:** 1 Hospital Geral Dr. Waldemar Alcântara, Departamento de Clínica Médica, Fortaleza, CE, Brasil.; 2 Hospital Geral Dr. Waldemar Alcântara, Fortaleza, CE, Brasil.; 3 Hospital Geral de Fortaleza, Departamento de Nefrologia, Fortaleza, CE, Brasil.; 4 Universidade Christus, Fortaleza, CE, Brasil.; 5 Universidade Federal do Ceará, Departamento de Clínica Médica, Programa de Pós-Graduação em Ciências Médicas, Fortaleza, CE, Brasil.

**Keywords:** Rapidly Progressive Glomerulonephritis, Post-infectious Glomerulonephritis, Leprosy, Staphylococcus, Glomerulonefrite Rapidamente Progressiva, Glomerulonefrite Pós-infecciosa, Hanseníase, Staphylococcus

## Abstract

Rapidly progressive glomerulonephritis (RPGN) is a renal disease with an extensive differential diagnosis. This paper reports the case of a 55-year-old female patient diagnosed with Hansen's disease with acute progressive renal impairment after developing lower limb pyoderma. The association between Hansen's and kidney disease has been well documented, with glomerulonephritis (GN) ranked as the most common form of renal involvement. Post-infectious glomerulonephritis (PIGN) in adults has been associated with a number of pathogens occurring in diverse sites. The patient described in this case report had RPGN and biopsy findings suggestive of PIGN with C3 and IgA detected on immunofluorescence and kidney injury secondary to recent infection by *Staphylococcus*, a well-documented manifestation of renal impairment in patients with Hansen's disease.

## RESUMO

A Glomerulonefrite Rapidamente Progressiva (GNRP) é um padrão de doença renal com amplo diagnóstico diferencial. O caso reporta uma paciente de 55 anos com deterioração aguda e progressiva da função renal após quadro de piodermite em membro inferior com diagnóstico concomitante de hanseníase. Associação da hanseníase com doença renal é bem descrita, sendo a GN a forma de acometimento renal mais comum. As glomerulonefrites pós-infecciosas (GNPIs) em adultos ocorrem devido a um grande número de patógenos, nos mais diversos sítios. A paciente do caso relatado apresentava quadro de GNRP e achados de biópsia que sugerem GNPI com marcação de C3 e IgA na imunofluorescência, sugestiva de lesão renal secundária a infecção recente por Staphylococcus, uma manifestação bem descrita de doença renal em pacientes com hanseníase.

## CASE REPORT

**Figure 1:**
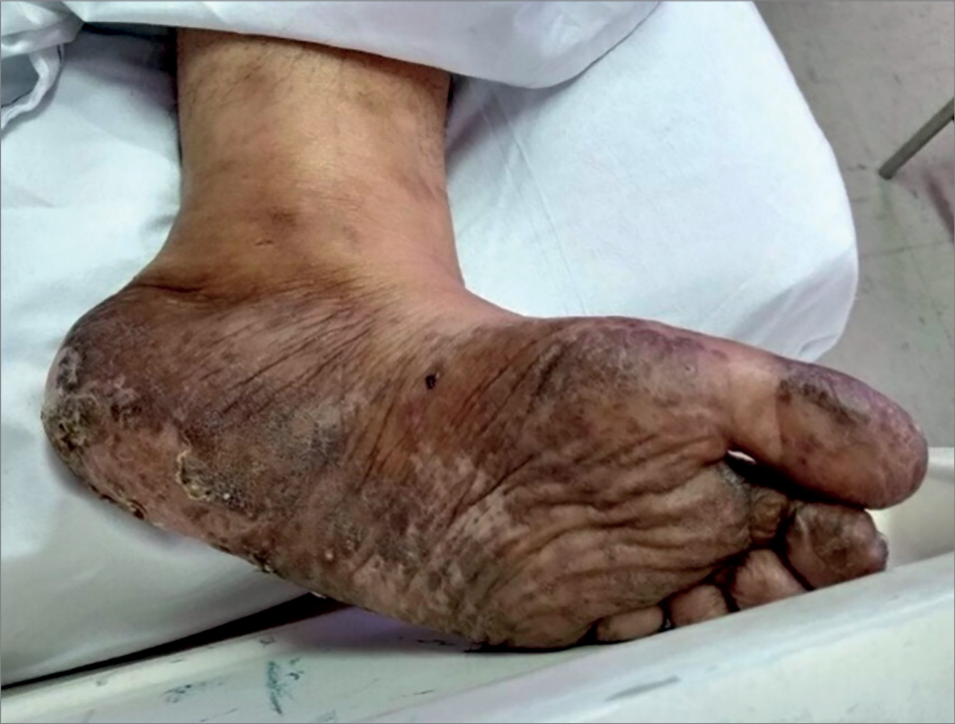
Hyperchromic lesions with altered sensitivity more evident on the soles of the feet.

A previously healthy 55-year-old female without known comorbidities was admitted to the General Practice Clinic of Hospital Geral Dr. Waldemar Alcântara (HGWA). She complained of weakness, paresthesia, and a burning sensation in her lower limbs she had been feeling for three years along with macular hyperchromic lesions on the soles of her feet. The patient went to a dermatologist nine months prior to admission and was diagnosed with contact eczema. She was prescribed topical corticosteroids and a moisturizing agent. One month before hospitalization the patient had pain, hyperemia, and bullous lesions on her right foot, which ruptured spontaneously letting out a serous secretion. She improved after taking unspecified medication. Five days prior to admission the patient developed oliguria, lower limb edema, and abdominal pain - mainly in the hypogastrium - along with nausea and hyporexia. She went to an Emergency Unit and was found to have a serum creatinine (SCr) level of 21.94 mg/dL and a blood urea nitrogen (BUN) level of 260 mg/dL, which triggered her referral to the hospital cited above. A test run six months prior to her arrival at the hospital read SCr = 0.7 mg/dL and blood urea= 37.4 mg/dL. Upon admission, she was found to be generally well and hydrated, pale 2+/4+, eupneic, conscious and oriented. Her heart was normal on auscultation while crackles were heard bilaterally on the bases of her lungs. She had a flaccid distended abdomen on account of fat accumulation and complained of pain on her hypogastrium upon palpation. No evidence of visceromegaly was found. Her peripheral pulses were palpable, and she had lower limb edema 1+/4+ and hyperchromic scar tissue-like lesions on the soles of her feet (Figure 1). Examination of the upper limbs revealed the interosseous muscles of her right hand were atrophied. Neurological examination showed she had predominantly distal paresis of the lower limbs (grade IV on the left and III on the right leg), grade IV paresis of the upper limbs, and anesthesia on the soles of her feet.

Lab tests performed upon admission (Table 1) were negative for HIV, syphilis, and hepatitis B and C. Tests for cytoplasmic (c-ANCA) and perinuclear (p-ANCA) antineutrophil cytoplasmic antibodies were negative; ANCA was atypical; the test for cryoglobulins was negative. Serum protein electrophoresis showed polyclonal increases of alpha-1-globulin and gamma globulins. Ultrasound examination of the kidneys and urinary tract showed normal-sized kidneys with irregular contours (RK: 9.1 x 4.3 cm, LK: 9.2 x 5.0 cm) and good corticomedullary differentiation. Transthoracic echocardiogram showed good cardiac function (EF 61%) and no vegetation. Fat pad biopsy was negative for amyloidosis. Electroneuromyography revealed distal mixed axonal demyelinating sensorimotor polyneuropathy with a predominant axonal component and preferential involvement of the right leg, producing severe impairment of the lower limbs and moderate to mild dysfunction of the upper limbs, as seen in cases of infectious neuropathies (including Hansen's disease), uremia, and vasculitis.

**Table 1:** Lab Tests on Admission

Hemoglobin (mg/dL)	6.8	Blood urea (mg/dL)	260
Hematocrit (%)	20.9	Creatinine (mg/dL)	21.94
VCM (fL)	90.9	TP/INR	1.31
CHCM g/dL	32.5	APTT	1.59
White blood cells (mm^3^)	8800	Sodium (mmol/L)	136
Segmented (%)	73%	Potassium (mmol/L)	6.1
Rods (%)	1%	Erythropoietin	7.5
Lymphocytes (%)	16%	PTH	107
Platelets (mil/mm^3^)	221	C3 (88 - 201)	84
CRP	5.66	C4 (VR: 16 - 47)	28
LDH	269	ANA	1/80; fine dotted pattern.
Urine test summary	citrine, slightly cloudy; pH = 6.5, density 1015, nitrite 1+, protein 3+, hemoglobin 3+, ketone bodies 1+, leukocytes 21/field; numerous red blood cells (with dysmorphism), moderate bacteriuria, gram-negative bacilli		

**Figure 2:**
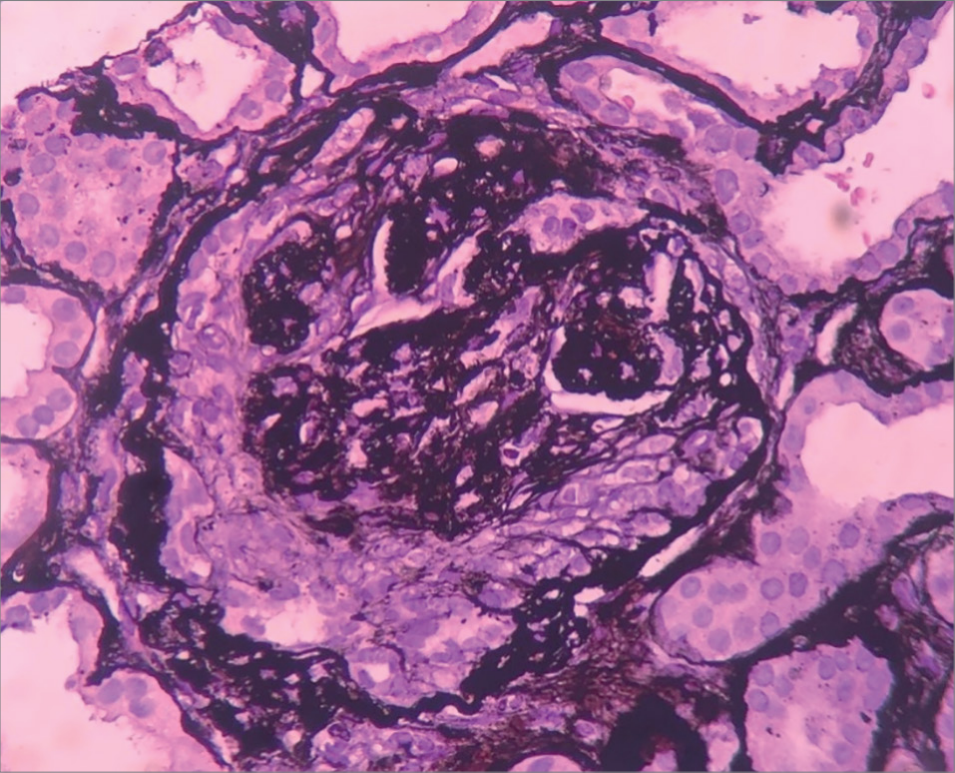
Light microscopy image of a kidney biopsy fragment - periodic acid silver methenamine stain (400x magnification) - showing a glomerulus with a fibro-cellular crescent.

**Figure 3:**
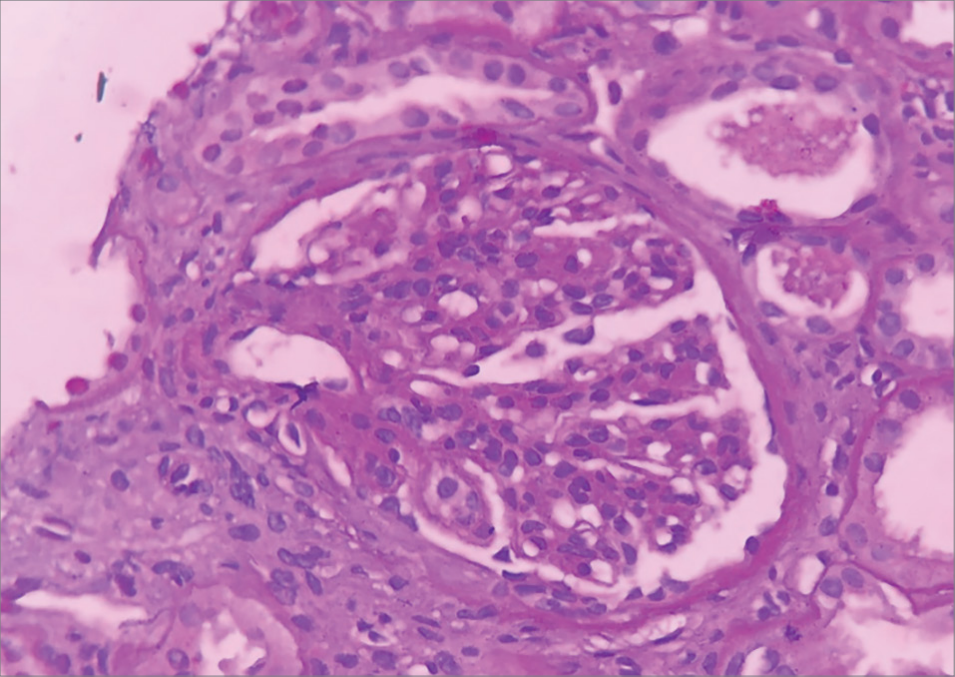
Light microscopy image of a kidney biopsy fragment - hematoxylin and eosin stain (400x magnification) - showing a glomerulus with mesangial proliferation.

**Figure 4:**
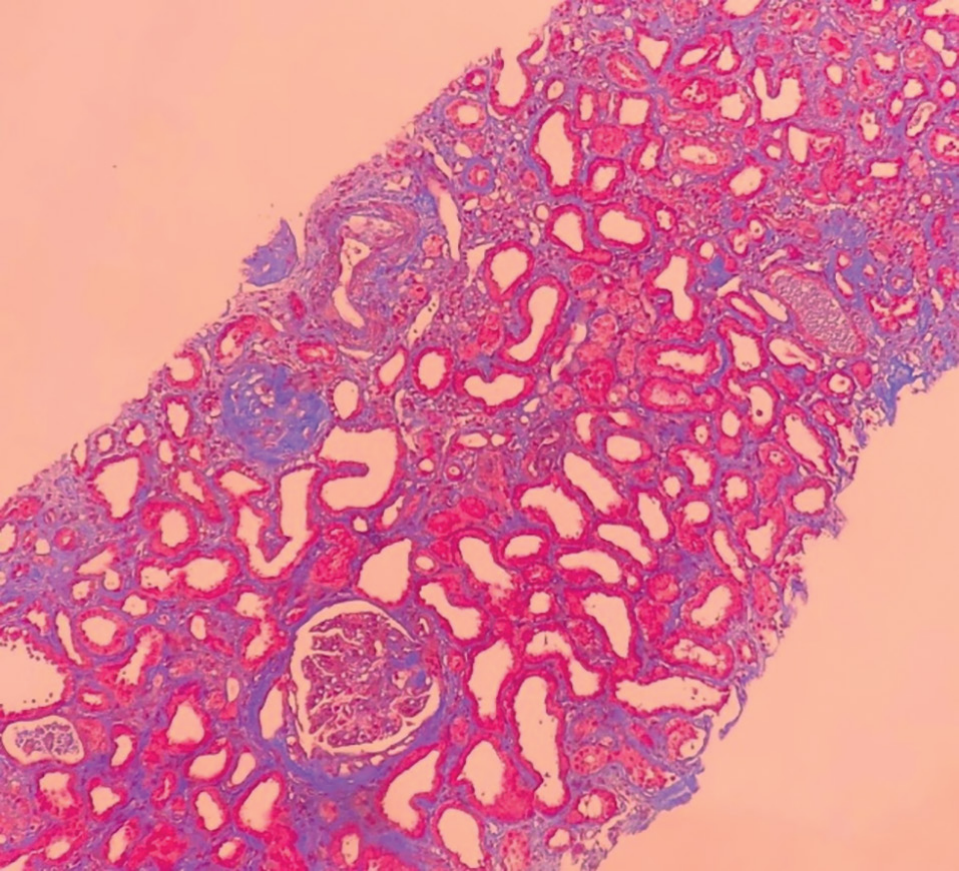
Light microscopy image of a kidney biopsy fragment - Masson's trichrome stain (100x magnification) - showing mild interstitial fibrosis.

**Figure 5:**
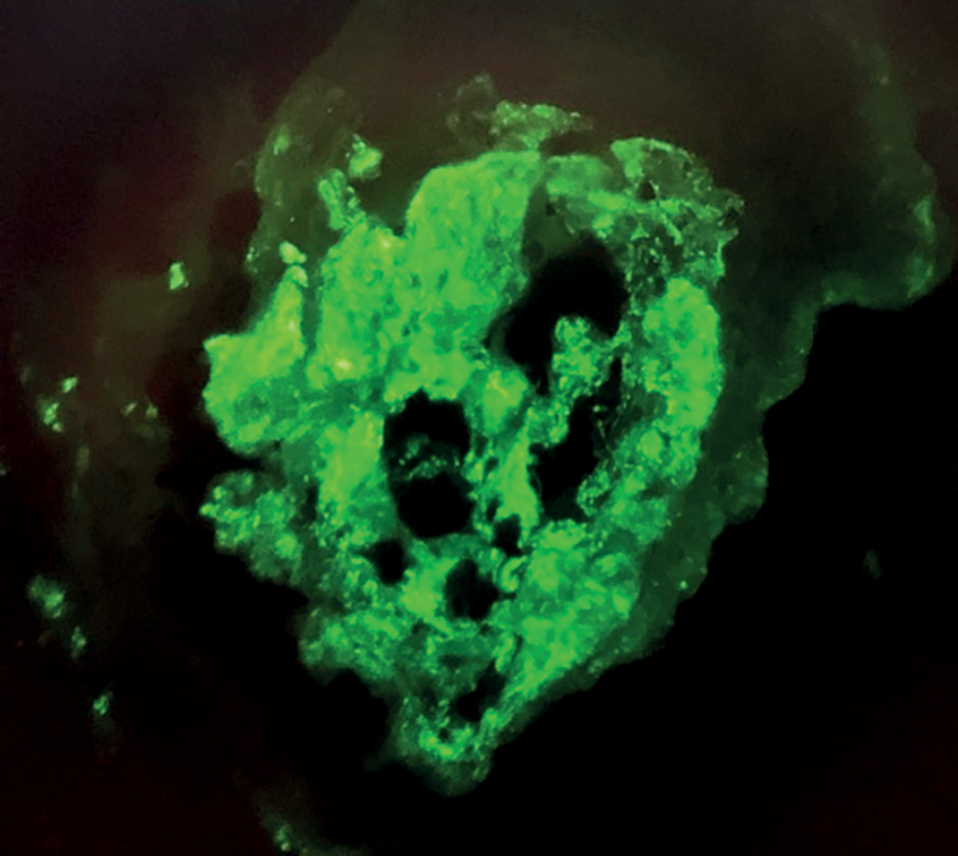
Immunofluorescence of kidney biopsy fragment showing strong labeling for C3 (400x magnification).

**Figure 6:**
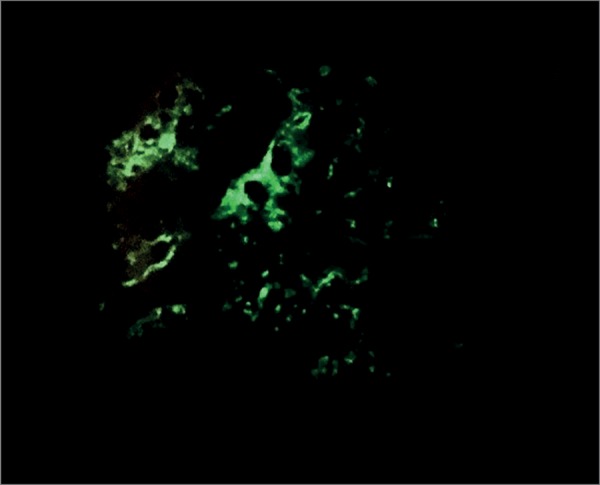
Immunofluorescence of kidney biopsy fragment showing strong labeling for IgA (400x magnification).

The patient was started on hemodialysis three times a week. Neurological assessment showed she had multibacillary Hansen's disease (positive for bacilli agglomerates). The patient was prescribed polychemotherapy with rifampicin, dapsone, and clofazimine. The choice was made to prescribe prednisone 1mg/kg/day two weeks after the start of treatment for Hansen's disease, since the patient had signs consistent with RPGN; she was waiting to undergo a kidney biopsy, which was performed only after 27 days of steroid therapy. The pathology specimen was satisfactory and featured 22 glomeruli and two medium-caliber vessels. Ten glomeruli had global sclerosis and three had fibro-cellular crescents (Figure 2). The other glomeruli had mild mesangial proliferation (Figure 3); findings such as polymorphonuclear infiltration and subepithelial or mesangial deposits (humps) were not seen. Mild to moderate interstitial fibrosis (Figure 4), acute tubular necrosis, and benign nephrosclerosis were also described. Immunofluorescence showed strong and diffuse labeling for C3 (3+ in 4+) in the mesangium, and barely positive results for IgA (1+ in 4+) in the mesangial compartment, following a pattern similar to that of C3 (Figures 5 and 6).

## DISCUSSION

The patient described in this case report suffered from significant loss of renal function within less than three months and had evidence of glomerular injury, hematuria, and proteinuria, which combined yielded a diagnosis of rapidly progressive glomerulonephritis, later confirmed by its pathological correspondent (crescentic glomerulonephritis). RPGN is caused by three disease groups: 1) Goodpasture syndrome or anti-glomerular basement membrane antibody disease; 2) pauci-immune glomerulonephritis; and 3) immune complex glomerulonephritis. Goodpasture syndrome stems from the presence of antibodies targeting the alpha-3 chain of type IV collagen of the GBM, and may manifest as a lung-kidney syndrome marked by linear deposition of IgG on the GBM confirmed by biopsy. Pauci-immune glomerulonephritis is characterized by the presence of antineutrophil cytoplasmic antibodies (ANCA), with p-ANCA (anti-myeloperoxidase) occurring more commonly in Churg-Strauss syndrome (eosinophilic granulomatosis with polyangiitis) and microscopic polyangiitis, while c-ANCA (anti-proteinase 3) is seen in Wegener's granulomatosis (granulomatosis with polyangiitis). Immune complex glomerulonephritis may be categorized as normocomplementemic (Henoch-Schönlein purpura, IgA nephropathy, and fibrillary GN) or hypocomplementemic (SLE, post-infectious GN, membranoproliferative GN, shunt nephritis, endocarditis, and visceral abscesses - predominant consumption of C3; cryoglobulinemia - predominant consumption of C4).^1^^,^ 2 ^,^ 3 ^,^ 4 ^,^ 5

The patient was diagnosed with Hansen's disease after she was found to be positive for bacilli agglomerates, a trait used to categorize the disease as multibacillary according to the World Health Organization. Hansen's disease is a chronic granulomatous infection caused by *Mycobacterium leprae,* a highly infectious pathogen that produces low morbidity.^6^ The association between Hansen's and renal disease has been well documented in the literature in the form of manifestations of glomerulonephritis, tubulointerstitial disorders, and chronic kidney disease with secondary amyloidosis.^7^^,^ 8 Renal impairment was found in 3.8% of the individuals enrolled in a large cohort study conducted by Daher *et al*., with the following associated main risk factors: episodes of lepra reaction (erythema nodosum in particular), multibacillary disease, male gender, age, and time with the disease. Several urinary alterations have also been described (proteinuria in 4.8%; hematuria in 6.8%; leukocyturia in 10.4%).^9^ Glomerulonephritis is the most common form of renal involvement, with no specific histopathology finding. Immunohistochemistry methods have identified granular deposits of IgG and C3, while IgA, IgM, and fibrin in the glomerular mesangium and capillaries have been reported less frequently. Patients with lower limb ulcers and altered sensitivity are more susceptible to secondary infection and, therefore, have a greater chance of developing post-infectious glomerulonephritis. The treatment of Hansen's disease with polychemotherapy and of lepra reactions with prednisone and thalidomide seems to improve renal function, particularly in patients with erythema nodosum leprosum.^7^^,^ 10

In the past, most of the cases of post-infectious glomerulonephritis (PIGN) were seen in children after skin or respiratory infection by *Streptococcus*. Prevalence in adults - the elderly and individuals on immunosuppressants in particular - is well documented and is on the rise.^11^ PIGN in adults can be caused by a number of pathogens and affect a wide array of sites (skin, upper airways, lungs, bones, heart, oral mucosa, teeth, and urinary system). It is more prevalent in males (3:1) and manifests, as in children, in the form of nephritic syndrome (hematuria, proteinuria, hypertension, and renal failure) usually 1-6 weeks after infection (sometimes infection along with kidney injury is suspected because the symptoms of infection are milder or less specific in elderly or diabetic individuals). Contrary to children, who rarely need dialysis, nearly half of the elderly patients are prescribed hemodialysis on account of uremic or congestive symptoms.^11^^,^ 12

In lab tests, it is characterized by complement consumption (C3 predominantly). Kidney biopsy is required for most adults suspected for PIGN to confirm the diagnosis and rule out glomerulonephritis with similar clinical presentation and for individuals in need of specific immunosuppressant therapy. PIGN is characterized by neutrophil-rich diffuse proliferative exudative glomerulonephritis. Crescents may form, but are less frequent in cases of pauci-immune GN. Immunofluorescence detects mainly the presence of C3 and possibly IgA in specific cases. Electron-dense subepithelial deposits ("humps") may be seen in electron microscopy images if pathology tests are not conclusive after correlation with clinical signs. Coupled with clinical history, these findings allow the discrimination of PIGN vis-à-vis other conditions considered in differential diagnosis (IgA nephropathy, Henoch-Schönlein purpura, ANCA-associated vasculitis). Satoskar *et al*. reviewed biopsies of patients with PIGN by *Staphylococcus* and found positivity for ANCA in 22% of tested patients and frequent detection of IgA in varied degrees of intensity (predominantly from mild to moderate).^13^^,^ 14 ^,^ 15

Treatment is based on the eradication of infection (antibiotics and surgery) and the management of nephritic syndrome (diet, antihypertensive medication, and diuretics). The role of immunosuppressants in PIGN is unclear, and this class of medications is not generally indicated. They may be prescribed to patients with PIGN (without evidence of active infection) with necrotizing and crescentic GN, and particularly to individuals with high ANCA titers.^12^

The patient described in this case report had glomerulonephritis with complement consumption (C3) and biopsy findings suggestive of advanced (chronic) post-infectious GN with C3 and IgA labeling on immunofluorescence, in addition to fibro-cellular crescents, glomerular sclerosis, and interstitial fibrosis. In this stage of the disease the characteristic subepithelial humps are less visible and involvement is essentially mesangial. The patient had signs suggestive of skin infection on her right foot before the onset of the renal symptoms associated with IgA labeling on immunofluorescence. Therefore, she may have had PIGN by *Staphylococcus*, a well-documented manifestation of renal disease in patients with Hansen's disease. The patient is still on dialysis and is currently weaning from glucocorticoids.
